# A case report of gastrointestinal hemorrhage caused by small intestinal hemangioma

**DOI:** 10.1097/MD.0000000000034526

**Published:** 2023-07-28

**Authors:** Yule Wei, Nan Yi, Yezhen Mo, Yunxiao Liang

**Affiliations:** a Department of Gastroenterology, The People’s Hospital of Guangxi Zhuang Autonomous Region, Guangxi Academy of Medical Sciences, Nanning, China.

**Keywords:** double balloon enteroscopy, gastrointestinal hemorrhage, small intestinal hemangioma

## Abstract

Small intestinal hemangioma is a rare condition and very difficult to diagnose preoperatively. It can occur in all segments of the small intestine, but jejunum is common. Its common symptoms are gastrointestinal bleeding and chronic anemia, while intussusception, intestinal obstruction and perforation are rare. In recent years, the popularization and application of capsule endoscopy, computed tomographic enterography and double-balloon enteroscopy play vital roles in the diagnosis and management of small bowel bleeding. We report a case of gastrointestinal hemorrhage caused by of the small intestine hemangioma.

**Patient concerns:**

A 56-year-old male complaint of hematochezia for 1 day with dizziness, fatigue, and vomiting of gastric contents.

**Diagnosis:**

Based on the clinical, laboratory, imaging tests, endoscopy, laparoscopic approach and pathological examination, the patient was diagnosed with small intestinal hemangioma.

**Interventions:**

Segmental resection was performed for the small intestinal hemangioma by a laparoscopic approach.

**Outcomes:**

The patient was discharged without operation complications, and his hemoglobin increased to 130 g/L at the second month after the operation.

**Lessons:**

Small intestinal hemangioma is a rare condition without specific symptoms and can cause gastrointestinal bleeding. The possibility of small intestinal hemangioma should be considered with unexplained gastrointestinal bleeding. Surgical resection is the preferred treatment option for symptomatic hemangiomas. Furthermore, double-balloon enteroscopy can increase the diagnostic yield. Applying endoscopic titanium clip combined with Indian ink marking can obtain an accurate positioning before surgery.

## 1. Introduction

Small intestinal hemangioma accounts for 7% to 10% of all benign small intestinal tumors.^[1]^ It can occur in all segments of the small intestine, but jejunum is common. Its common symptoms are gastrointestinal bleeding and chronic anemia,^[2]^ while intussusception, intestinal obstruction and perforation are rare. In recent years, the popularization and application of capsule endoscopy, computed tomographic enterography and double-balloon enteroscopy play vital roles in the diagnosis and management of small bowel bleeding. This report describes a patient who presented with massive gastrointestinal hemorrhage caused by of the small intestine hemangioma. Informed written consent was obtained from the patient for publication of this report.

## 2. Case presentation

**Figure 1.**
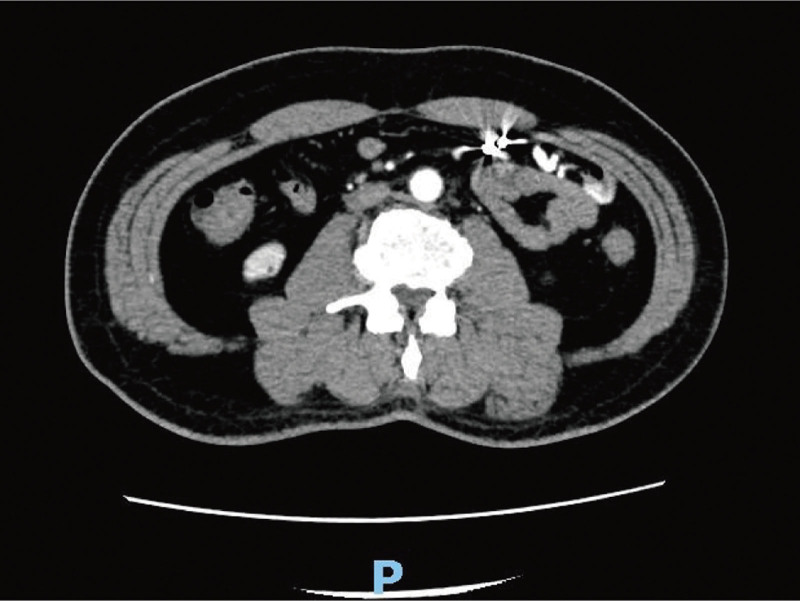
Vascular malformation of the small intestine in the left upper quadrant was shown by abdominal contrast-enhanced computed tomography (CT).

**Figure 2.**
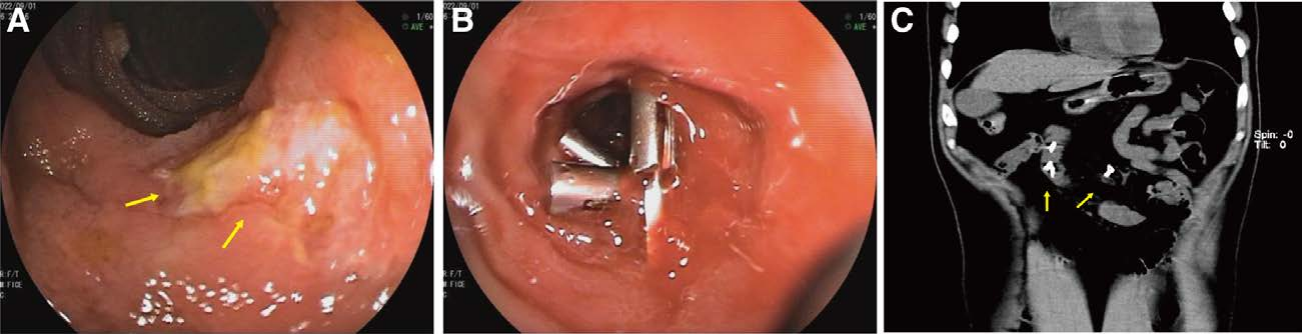
(A–B) Jejunal ulcer was indicated by double-balloon enteroscopy, witch clamped with 3 titanium clips; (C) Abdominal computed tomography showed titanium clip localization.

**Figure 3.**
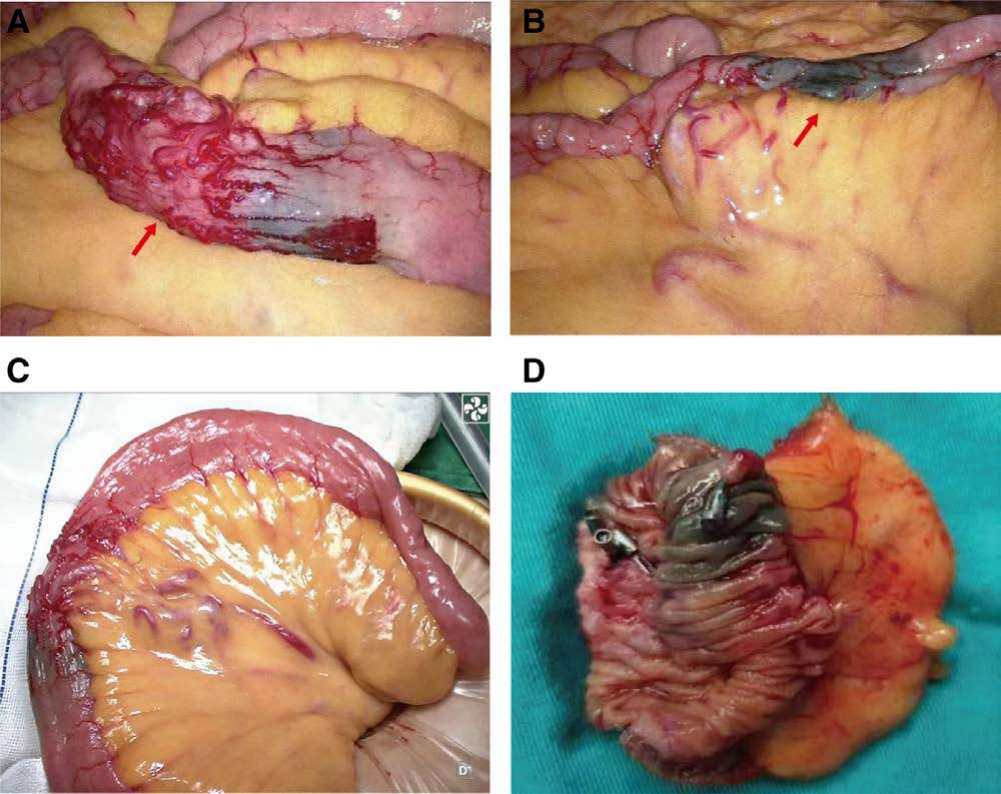
(A–D) Intraoperative appearance showed the diseased bowel and mesenteric vessels with localization by India ink.

**Figure 4.**
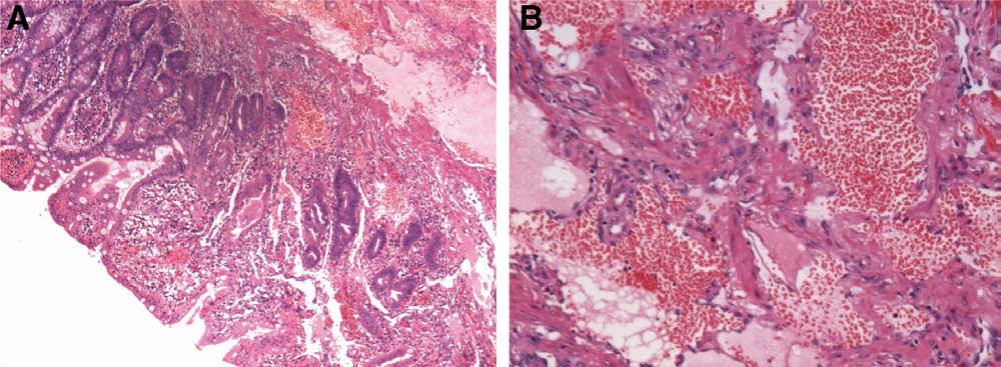
(A and B) Histologic section of the resected specimen identified as hemangioma of the small intestine. The field magnification of microscopic is 100×.

A 56-year-old male complaint of hematochezia for 1 day with dizziness, fatigue, and vomiting of gastric contents. The amount of bleeding is approximately 1200 mL. Emergency laboratory tests revealed low hemoglobin level of 5.2 g/dL. He didn’t vomit blood or have abdominal pain. His past medical history included embolization for hemorrhage from ruptured superior mesenteric artery branches, iron deficiency anemia, gastric ulcer, antral gastritis, and he referred to recent use of acetamidophenol. No personal or family history of gastrointestinal malignancies was present. On physical examination, the patient had pale conjunctivae, the abdomen was soft, not distended, and non-tender. Laboratory tests hypo chromic anemia, normal tumor markers. Contrast-enhanced computed tomography of the abdomen demonstrated vascular malformation of the small intestine in the left upper quadrant (Fig. 1). Upper gastrointestinal endoscopy detected ischemic mucosa, but without active bleeding lesion or tumor. The colonoscopy did not detect significant abnormalities other than internal hemorrhoids and ileocecal valve polyp. Double-balloon enteroscopy revealed jejunal ulcer and suspicious multiple lymphangiectasia. The ulcer was clamped with 3 titanium clips and marked with India ink (Fig. 2A–C). For a definite diagnosis, the patient underwent a laparoscopic exploratory. During the operation, approximately 10 cm diseased bowel which be stained with Indian ink was found at 150 cm distal to the Treitz ligament. The vessels of the diseased bowel and the mesenteric were significantly dilated and tortuous. In addition, multiple telangiectasia in the small intestine and massive blood accumulation in the colon can be seen. The diseased bowel was resected and a side-to-side anastomosis was performed (Fig. 3A–D). Pathological examination revealed erosion of small intestinal mucosa, vascular proliferation was found in the whole layer of the intestinal wall, all of which are consistent with small intestine hemangioma (Fig. 4A–B).

## 3. Discussion

Most types of vascular disease are caused by germ cell or somatic mutations during the embryonic period. Small intestinal hemangioma is a kind of congenital abnormal developmental disease, which comes from submucosal vascular plexus and involves mucosa, muscularis, and mesentery. Histologically, hemangioma was classified as capillary hemangioma, cavernous hemangioma and mixed hemangioma. Among them, cavernous hemangioma is the most common,^[3]^ which can infiltrate large bowel and mesentery. It often occurs in the jejunum, can be single or multiple.

Small intestinal hemangioma lacks specificity in clinical manifestations, and early diagnosis is difficult due to the long length of small intestine and certain range of motion. The main examination methods of small intestinal hemorrhage^[4,5]^ include whole digestive tract barium meal angiography, small intestine angiography, digital substraction angiography, emission computed tomography, capsule endoscope and enteroscopy, etc. Capsule endoscopy is a noninvasive examination method, which has a high diagnosis rate in the aspects of dominant and persistent bleeding, but in the case of acute bleeding, the observation will be affected due to poor visual field. At present, it still has some limitations, such as inaccurate positioning, inability to take a tissue biopsy and capsule retention. Enteroscopy is intuitive and operability technique, which allowing for biopsy and endoscopic treatment. It has important clinical value in preoperative localization of small intestinal bleeding. However, it requires a high level of skill from the doctors, and has the risk of intestinal bleeding and perforation, and is poorly tolerated by patients. In recent years, domestic reports on small intestinal hemangioma are mainly case reports, most of which are retrospective studies of enteroscopy and capsule endoscopy.^[6–11]^ The patient was admitted to our hospital due to gastrointestinal bleeding. There were no obvious signs at the time of onset, and no bleeding lesions were detected by gastroscopy and colonoscopy. However, enteroscopy showed jejunal ulcer, but no intestinal stenosis, blood vessel exposure, bleeding and other phenomena. Based on the comprehensive analysis of the condition and imaging findings, it is more likely to be diagnosed as small intestinal hemangioma or vascular malformation. According to the report, the incidence of later rebleeding by angiography embolization in the treatment of lower gastrointestinal bleeding is 22%,^[12]^ while the rate of rebleeding in treatment of vascular dilatation is as high as 40%.^[13]^ The patient underwent angiographic embolization 4 years ago for a ruptured superior mesenteric artery branch. If the same treatment is given this time, there may be a risk of rebleeding. And considering that his condition is deteriorating and causing massive blood loss, which could be life-threatening. In view of this, the ulcer area of the lesion was small and lacked tactile sensation, so titanium clips were used to clamp the ulcer. Titanium clips can be used for X-ray or computed tomography imaging to indicate the location of the lesion, can be felt by hand during open surgery, and can avoid the risk of transmural injury and perforation.

In order to facilitate laparoscopic exploration, the lesions were stained with Indian ink. The dyeing technique is non-dispersive, durable, safe and effective.^[14]^ The combination of these 2 methods provides accurate positioning for laparoscopic exploration and minimally invasive surgery, thus effectively exposing the surgical field and saving the operation time.

In this case, it was found that the titanium clip location and Indian ink stain section were all located in the jejunum under laparoscopy, and a large number of meandering blood vessels could be seen here, winding and extending to the mesentery. Overall, radical resection of the diseased bowel is the best plan.

## 4. Conclusion

In conclusion, due to the lack of specificity in clinical manifestations of small intestinal hemangioma. The possibility of small intestinal hemangioma should be vigilant when encountering symptoms of unexplained gastrointestinal bleeding. In this case, colonoscopy is of high diagnostic value. With the help of the colonoscope, we closed the lesions using titanium clips and achieved precise guidance through the Indian ink staining technology, creating favorable conditions for subsequent minimally invasive surgical treatment.

## Author contributions

**Conceptualization:** Yunxiao Liang.

**Data curation:** Yule Wei, Yezhen Mo.

**Funding acquisition:** Yule Wei, Yunxiao Liang.

**Project administration:** Yunxiao Liang.

**Supervision:** Yunxiao Liang.

**Writing – original draft:** Yule Wei.

**Writing – review & editing:** Yule Wei, Nan Yi, Yunxiao Liang.
